# Impact of tropical cyclones on child marriage rates in 14 affected countries

**DOI:** 10.1088/2752-5309/aead12

**Published:** 2026-10-12

**Authors:** Anna Y Palmer, Yuta J Masuda, Aurélie P Harou, Margaret E Greene, Jai K Das, Christina T Kwauk, Brian E Robinson, Alissa Koski, Jill Baumgartner

**Affiliations:** 1 Department of Epidemiology, Biostatistics and Occupational Health, McGill University, Montreal, Canada; 2 McGill Centre for Climate Change and Health, McGill University, Montreal, Canada; 3 Allen Family Philanthropies, Washington, Seattle, United States of America; 4 Department of Natural Resource Sciences, McGill University, Montreal, Canada; 5 GreeneWorks, Washington, DC, United States of America; 6 Institute for Global Health and Development, Aga Khan University, Karachi, Pakistan; 7 Division of Women and Child Health, Aga Khan University, Karachi, Pakistan; 8 Unbounded Alliance, University Park, Maryland, United States of America; 9 Department of Geography, McGill University, Montreal, Canada; 10 Department of Equity, Ethics, and Policy, McGill University, Montreal, Canada

**Keywords:** child marriage, tropical cyclones, tropical storms, climate change, gender-based violence

## Abstract

Tropical cyclones (TCs) have had reported impacts on child marriage within certain communities, driven by disrupted livelihoods, displacement, and interruptions to education. However, the extent to which TCs affect national rates of child marriage has not been systematically studied. Here, we combine 12 888 607 person-years of nationally representative survey data on child marriage with high-resolution gridded wind speed data to estimate the effect of TCs on child marriage rates in 14 cyclone-exposed countries between 1983 and 2015. Using a distributed lag model, we estimate changes in child marriage rates up to three years following a cyclone. We find little measurable change in national child marriage rates after cyclones. We observe a small increase in the rate of child marriage in Mozambique, and subgroup analyses suggest that child marriage may have increased slightly among urban populations in Cambodia and Timor-Leste. Overall, these results indicate that, despite reported impacts, TCs are unlikely to be a dominant driver of child marriage rates globally.

## 1. Introduction

The world is increasingly likely to experience 2.6 °C–3.1 °Cof anthropogenic warming by the end of the century [1, 2], underscoring the urgent need to strengthen the resilience of vulnerable and marginalised populations. Tropical cyclones (TCs) are a key climate extreme expected to intensify under climate change, driven in part by sea-level rise and higher precipitation rates [3, 4]. TCs can stunt economic growth, causing decade-long reductions in per capita income [5, 6], and affect populations through multiple channels, including damage to infrastructure, food systems, and healthcare services [7]. While broad-scale evidence on TC impacts across populations (particularly in high-income countries) is now better characterised [6, 8–11], evidence of adverse effects elsewhere remains limited. This gap is especially pronounced for children in low- and middle-income countries, where vulnerability to TCs is highest [12, 13]. In these contexts, TCs have been linked to decreased school enrolment [14], child abuse [15], child labour [16], and lower test scores and educational attainment [17, 18].

One outcome that remains underexplored, however, is whether, and to what extent, TCs influence child marriage: an internationally recognised form of gender-based violence and global indicator of gender inequality [19]. Defined by the United Nations as marriage before age 18 [20], child marriage has significant consequences for health [21], education, and autonomy [22]. An estimated 640 million women and girls alive today were married as children [23]. Though many of the determinants of child marriage are structural and slow-moving [24], recent reports from major NGOs and qualitative studies have documented cases where acute events, such as TCs and flooding, have contributed to child marriage, driven mainly by disrupted livelihoods, displacement, and interruptions to education [25–29]. These accounts have raised concerns that climate change could become a significant driver of child marriage globally [28, 29] and have resulted in explicit calls for more quantitative research into how climate crises are affecting child marriages [30].

TCs cause a myriad of damages, and as a result, there are many reasons to believe they could acutely heighten household vulnerability in ways that influence the rate of child marriage. For instance, economic hardship following a cyclone may increase child marriage as families seek to reduce household expenses, secure marriage payments, or arrange marriages for their daughters into more stable households [26, 27, 31]. The direction of this economic channel may depend on the prevailing marriage-payment structure: where a bride price is paid to the bride’s family, hardship may accelerate marriage as a source of income [32], whereas where the bride’s family pays a dowry, the effect is ambiguous, since the cost may discourage marriage but also may encourage it if younger brides command lower dowries [26, 33, 34]. In communities where migration follows TCs, displacement and loss of social networks could further heighten risks, particularly if girls are perceived as vulnerable to sexual violence [25, 26]. Additionally, disruptions to education, one of the strongest protective factors against early marriage [35], may remove a critical barrier to child marriage, increasing rates in affected areas.

Recent reviews document some studies examining the relationship between climate change and child marriage [29, 36], though the extent to which acute climate shocks like TCs influence child marriage rates in the years immediately following exposure remains unclear. Studies quantitatively examining the impact of TCs and other climate extremes on child marriage rates have been limited to single-country contexts or have presented pooled estimates when conducting multi-country analyses [37]. Generating country-level estimates is critical not only for identifying vulnerable settings but also because these contextual factors may cause the response to TCs to differ across countries rather than produce uniform increases. In this study, we examined the response of child marriage rates to TCs in 14 affected countries over 32 years, operating in the years immediately following a cyclone. Using nationally representative survey data and high-resolution wind speed estimates, we estimated the effect of cyclone exposure on child marriage rates up to three years post-landfall, presenting separate effects for each country. We also examined whether the effects vary across rural/urban populations, in different time periods, and with different definitions of exposure.

## 2. Methods

### 2.1. Child marriage data

We used data from the Demographic and Health Surveys (DHS) [38], which are considered the most reliable sources of nationally representative data on child marriage [39]. Countries were included in the analysis if they (i) were exposed to TC winds between 1983 and 2015 and (ii) had at least one publicly available DHS survey (as of August 2025) with geocoded cluster coordinates. This yielded 14 countries (SI: table S1).

DHS employs a multistage sampling design, selecting census enumeration areas based on population size and stratification criteria (i.e. rural/urban status) and then 20–30 households within each enumeration area. Women aged 15–49 years report their marital history, including the month and year of their first marriage or the month and year they started cohabiting with a man as if married. DHS applies a multi-stage validation procedure and imputes missing marriage dates based on other reported information (such as reported year of birth, reported year of first child’s birth, and age of first sexual intercourse). Year and/or age of first marriage is reported for virtually all participants within DHS surveys (SI: figure S1). All data were anonymised.

Although DHS is cross-sectional, we used the data to reconstruct a longitudinal dataset using respondents’ reported birth and marriage years. For example, a woman interviewed in 2020, born in 1994 and married in 2009, is classified as unmarried at ages 10–14 and as married at age 15. We expanded the data so that each row represented a person-year from ages 10–17, with a binary indicator of marriage. Age 10 serves as the lower bound for being at risk of child marriage, as marriage before this age is rare [40]. The data are truncated the year after marriage, since individuals are no longer at risk of child marriage after that year.

### 2.2. TC data

We measured TC exposure using modelled wind speeds from the TCE-DAT database [41]. While TCs can cause damage through storm surges, intense rainfall, flooding, and high winds, we used wind speed as a proxy for overall exposure because it strongly correlates with these other hazards [8]. The TCE-DAT data are derived from TC track and intensity information in IBTrACS, the most comprehensive global database of TC observations [42], which it uses to model surface wind distributions. The resulting dataset provides one-minute maximum sustained wind speed (in knots) at a 0.1° × 0.1° grid resolution for each year and location affected by landfalling TCs recorded in IBTrACS between 1950 and 2015. To align with the availability of child marriage data, we restricted our analysis to the period 1983–2015.

We linked TC exposure estimates to the expanded panel of child marriage data using geocoded cluster coordinates from the DHS surveys. Each cluster was matched to the corresponding grid cell in the TCE-DAT dataset for every calendar year. For instance, an unmarried individual observed in 2000 was assigned the maximum wind speed recorded in the grid cell containing their cluster for that year. Individuals were classified as exposed to a TC in a given year if the maximum wind speed exceeded 34 knots, consistent with the Saffir–Simpson threshold for a tropical storm [43].

### 2.3. Empirical strategy

We leveraged the quasi-random nature of TC incidence to estimate its impact on child marriage rates, assuming that the effects of a TC on child marriage may persist in the years following TC exposure. Specifically, we employed the following fixed effects linear probability model:

\begin{equation*}{Y_{ijt}} = \alpha + \mathop \sum \limits_{l = 0}^3 {\beta _{cl}}\left( {{I_c} \times {W_{j,t - l}}} \right) + \lambda Ag{e_{it}} + {\delta _j} + {\gamma _{ct}} + {\varepsilon _{ijt}}\end{equation*}

where ${Y_{ijt}}$ indicates whether individual i in cluster j (in country c) was married in year t. ${W_{j,t - l}}$ is an indicator for whether cluster j was exposed to a TC l years prior, and ${I_c}$ is a country indicator used to estimate country-specific exposure effects. $Ag{e_{it}}$ denotes the individual’s age in year t. ${\delta _j}$ are cluster fixed effects, ${\gamma _{ct}}$ are country-year fixed effects, and ${\varepsilon _{ijt}}$ is the error term. Each ${\beta _{cl}}$, therefore, represents the effect of cyclone exposure *l* years prior, pooled across the within-cluster variation in exposure from all clusters in country *c*.

Our model estimates the causal effect of TCs on child marriage by comparing the likelihood of child marriage in exposed person-years to the likelihood of child marriage in unexposed person-years among person-years within the same cluster. By comparing exposed and unexposed person-years within the same cluster, our design isolates the short-run deviation in child marriage rates attributable to cyclone exposure, while differencing out the time-invariant structural characteristics that drive baseline differences in child marriage rates between clusters. Thus, the cluster fixed effects non-parametrically account for unobserved, cross-cluster differences, such as differences in cultural norms, religion, access to education, and urbanicity (SI figure S1). Because these characteristics tend to change only gradually, they can reasonably be treated as time-invariant within clusters over the study period and are captured by the cluster fixed effects without requiring separate adjustment. Country-year fixed effects control for nonlinear and discontinuous changes in child marriage rates common to all locations within a country, such as those caused by changes in child marriage laws and widespread conflict. We also adjust for age, since girls are significantly more likely to be married in their older teens [40]. Other important determinants of child marriage measured in DHS, such as years of schooling and wealth status, are not included as covariates since these variables were measured at the time of the survey rather than when individuals were at risk of child marriage and thus could have been a consequence of child marriage. Moreover, our empirical strategy controls for time-invariant factors that may influence child marriage by design, mitigating the potential for these factors to confound our analysis.

We present the ${\beta _{cl}}$ coefficients separately for each country, reflecting the expectation that the response to a cyclone is conditional on context, since the 14 countries in our sample differ in customary marriage payments, baseline child marriage rates, and disaster preparedness capacity. We include lags up to 3 years after TC landfall, as effects on child marriage are most likely to occur within 1–2 years of exposure [27]. Where the effects of TCs on child marriage may be overlapping in time (i.e. in cases where multiple TCs are observed within a 3 year period), our model assumes that there is a consistent response function across events, and that the overlapping effect is additive. Standard errors were clustered at the DHS cluster level, which is the unit at which TC exposure varies in our data [44].

### 2.4. Effect heterogeneity

Since rural and urban populations differ in population density, infrastructure, and livelihood sources, we assess heterogeneity in effects by interacting the ${\beta _{cl}}{\text{ }}$ coefficients with an indicator for rural–urban status. Using the same interaction approach, we also tested whether the relationship between TCs and child marriage has changed over time by interacting wind speed with an indicator for pre- and post-2000 periods, and present cumulative exposure–response curves for each time frame.

### 2.5. Sensitivity analyses

Because TC exposure was assigned based on women’s survey location rather than their location during the years they were at risk of child marriage, exposure status may be misclassified if women migrated between these two time points. In some surveys, DHS asks women whether they have always lived in the same city, town, or village, and, if not, how long they have lived in the same place. Although previous research shows that most marriage migration among women occurs over relatively short distances [32], we also conducted a sensitivity analysis restricting the analysis to women who reported never moving or who moved before age 10, where migration history data were available. Since this restriction could induce collider stratification bias if both child marriage and TC exposure influence migration [45], we also estimated whether TC exposure affected migration by re-estimating our main model with migration as the outcome. This allowed us to assess the reliability of the results from our sensitivity analysis.

To test the robustness of our findings to alternative exposure definitions, we also repeated the analysis using a stricter exposure definition of greater than 64 knots (the threshold for a Category 1 hurricane).

## 3. Results

Our data comprised 12 888 607 person-years obtained from 37 nationally representative surveys in 14 countries. The global distribution of TC exposure is shown in figure 1, along with the proportion of individuals in each DHS cluster married before the age of 18. Child marriage is prevalent across all 14 countries, with the lowest proportion of girls married before the age of 18 in the Philippines (20.9%), and the highest proportion in Bangladesh (76.9%). Though the global prevalence of child marriage has declined markedly over the last two decades [46], the annual probability of child marriage among 10–17 year olds in our sample remained relatively stable, with little major shifts over the period 1983–2015 (SI: figure S2).

**Figure 1.**
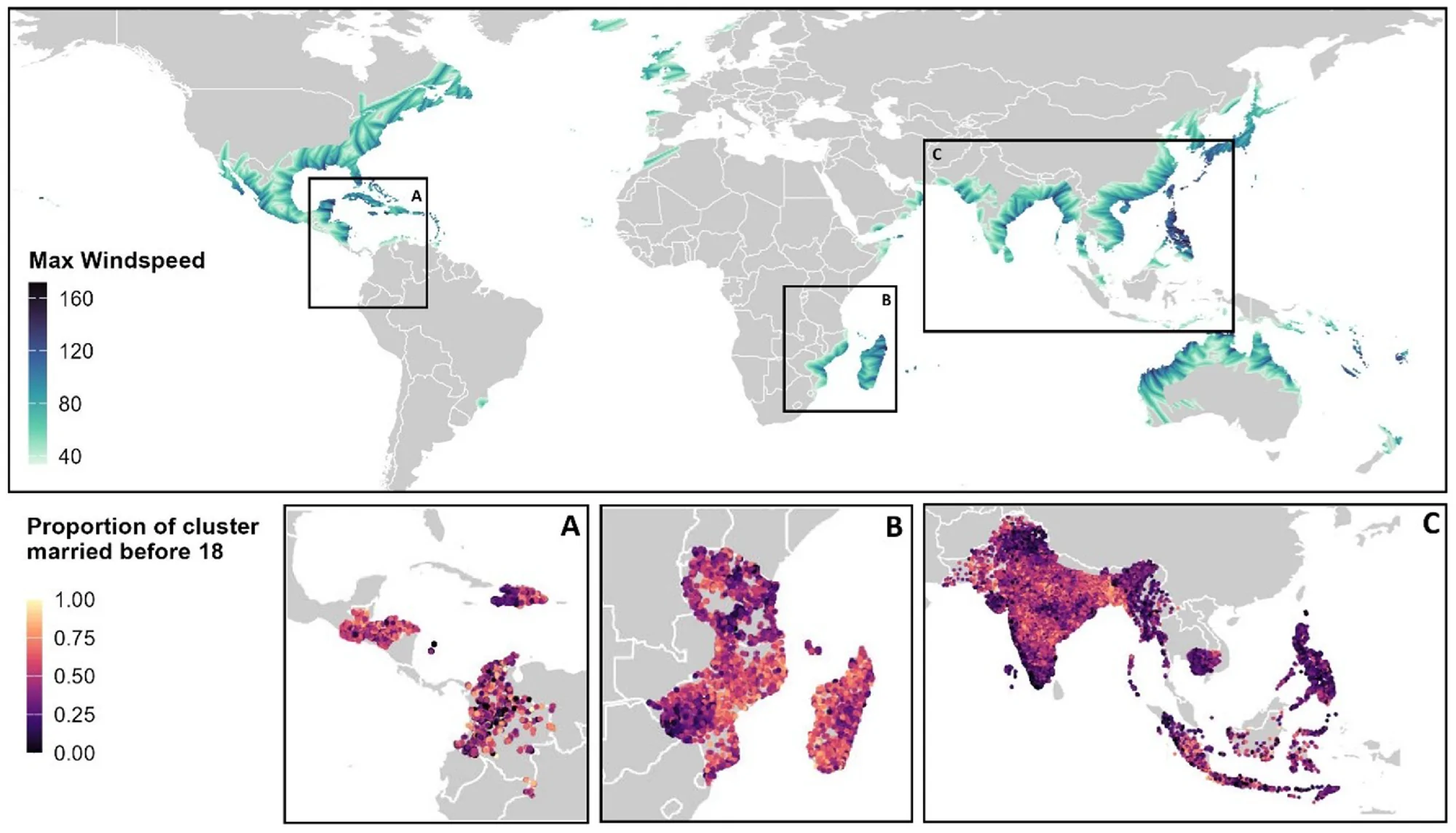
Map showing the global maximum windspeeds (in knots) within each 0.1° × 0.1° grid cell 1983–2015 based on TCE-DAT, and the proportion of each DHS cluster married before 18 in Dominican Republic, Guatemala, Haiti, Honduras (A), Comoros, Madagascar, Mozambique, Zimbabwe (B), Bangladesh, Cambodia, India, Myanmar, Philippines and Timor-Leste (C).

Approximately 13.6% of person-years across the entire sample were considered exposed to TCs, defined as experiencing wind speeds greater than 34 knots. However, this varied considerably by country. Exposure was most frequent in the Philippines, where 52.2% of person-years were considered exposed to TCs. Temporal patterns of cyclones also varied (SI: figure S3). For instance, in the Philippines, cyclones occurred on an almost annual basis, whereas in Bangladesh, cyclones were a temporally dispersed phenomenon.

We find that exposure to TCs had little measurable effect on the rate of child marriage (figure 2). In all countries, child marriage rates remained relatively stable up to three years following TC landfall. Mozambique showed a slight increase in the rate of child marriage during the year of TC landfall, corresponding to a one percentage point change in the rate of child marriage. Given a baseline child-marriage rate of 0.059 in Mozambique, the relative increase in the rate of child marriage corresponded to approximately 17%.

**Figure 2.**
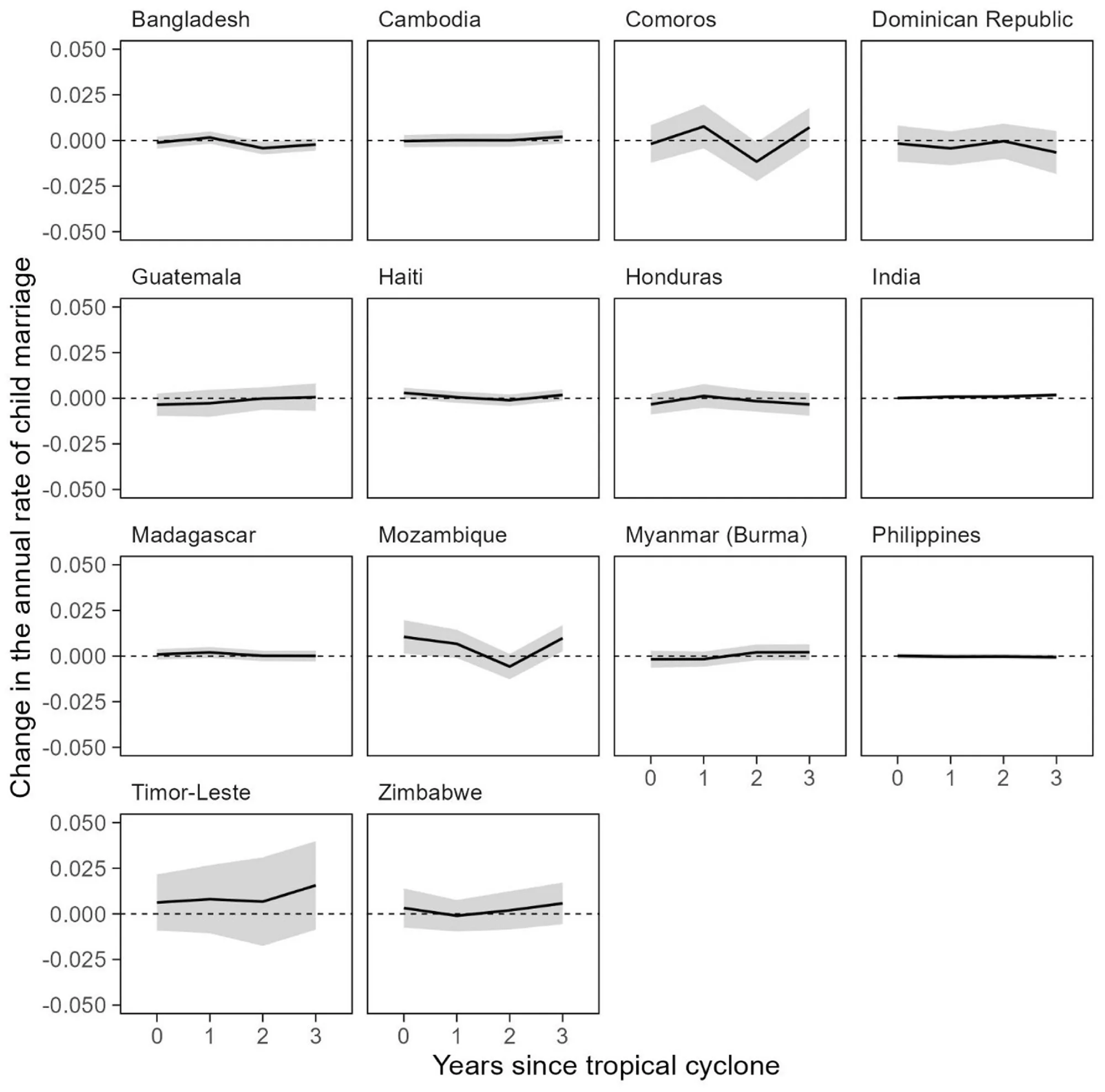
Estimated effect of tropical cyclone exposure (defined as being exposed to wind speeds greater than 34 knots) on the annual rate of child marriage, each year after TC landfall. Estimates are produced for each country annually.

We found little change in the effect estimates when defining exposure to TCs as experiencing >34 knots of wind compared to >64 knots of wind (figure 3). The effect of TCs also appeared similar between the pre- and post-2000 period in most countries. In Cambodia and Mozambique, the rate of child marriage appeared to increase within the post-2000 period, while in Honduras, child marriage appeared to increase within the pre-2000 period. Again, the effect within rural and urban populations was also similar within most countries. However, there was a slight increase in the rate of child marriage among urban populations in Cambodia and Timor-Leste.

**Figure 3.**
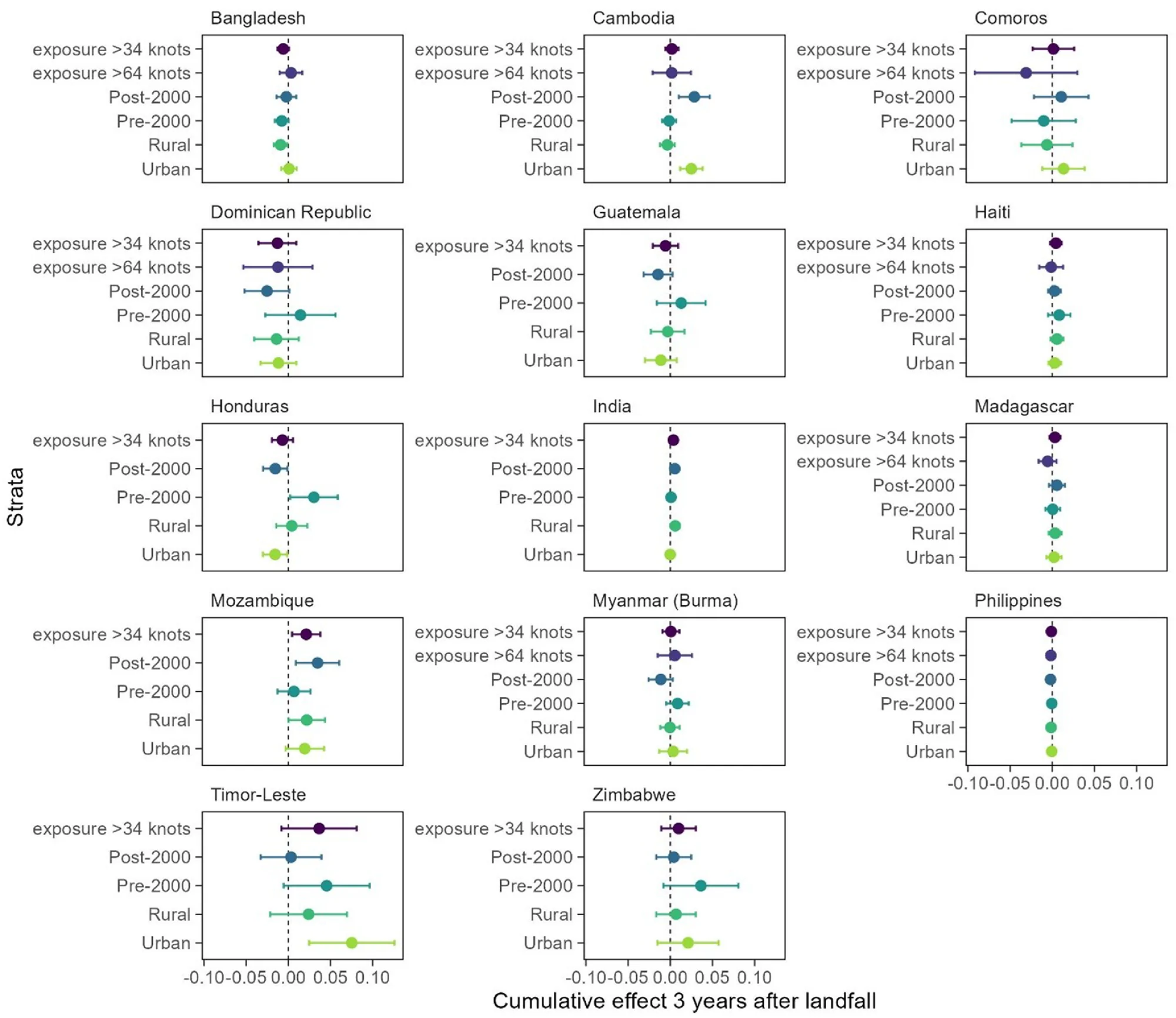
Estimated cumulative effects of tropical cyclones on annual child marriage rates, measured up to three years after landfall. Results are shown separately for each country under different exposure thresholds (winds >34 knots vs >64 knots), for pre-2000 and post-2000 periods, and for rural versus urban populations. For countries with limited exposure to winds >64 knots (Guatemala, Honduras, India, Mozambique, Timor-Leste, and Zimbabwe), estimates for this threshold are not presented.

Migration histories for women were collected within 10 of the 14 countries included in our analysis. When re-estimating the model using only data from women who reported always living in the same location or moving before the age of 10, we observe little change in the results (SI: figure S4). However, we observed a slight increase in child marriages in Zimbabwe in the third year after the TC. We also found that TC exposure was not associated with migration in the same year as the TC (SI: figure S5), which suggests that collider stratification bias in our sensitivity analysis was not a major concern.

## 4. Discussion

Although concern has grown that TCs may increase rates of child marriage, our findings suggest that TCs do not translate into measurable shifts in national child marriage rates in most countries in the years immediately following the cyclone. We observed a slight increase in Mozambique during the same year that the cyclone made landfall, as well as small increases among urban populations in Cambodia and Timor-Leste. Overall, the evidence suggests that while TCs may exacerbate risks in certain contexts, they are not a dominant driver of child marriage globally.

These results raise important questions around which factors are most influential in shaping child marriage rates globally. While current frameworks on child marriage emphasise the interplay of economic, cultural, social, and institutional factors [24, 47] the relative weight of these drivers remains unexplored. Environmental shocks such as TCs are often hypothesised to increase child marriage through economic hardship, but the limited effects observed here suggest that short-term economic shocks may not be a predominant driver in most contexts. This aligns with our prior research on droughts, a longer-term and more sustained environmental exposure, which similarly found little to no effect on child marriage rates in 61 countries [36]. Some research has shown that deeply embedded social and cultural norms play a far more decisive role in shaping marriage decisions than temporary economic disruptions [48, 49]. However, significant work remains to uncover the role of cultural and social norms in driving child marriage and to identify effective strategies for addressing them globally.

Although we find little evidence of a systematic relationship between TCs and child marriage at the population level, child marriage may still be occurring in response to extreme events within subsets of the population. Qualitative studies document instances where respondents reported that extreme weather events influenced household decisions around child marriage, particularly within households experiencing forced displacement, poverty, or food insecurity [26, 27, 31, 33]. If such cases remain relatively rare or limited to specific socio-demographic groups with high vulnerability, their effects may be too small to register in national estimates. Consistent with this, our analysis identifies some country-specific effects (in Mozambique, Cambodia, and Timor-Leste), albeit small in magnitude. These three settings share a custom of payment from the groom’s family to the bride’s family [50–52], under which marrying a daughter can generate liquidity after an income shock. In India and Bangladesh, which have the largest number of person-years in our sample, transfers run the other way and asset loss raises the cost of marrying a daughter. We treat this as a hypothesis rather than an explanation, since the pattern does not sort cleanly by direction of marriage payments, and we would expect some estimates to depart from the null by chance. Given the scale and increasing prevalence of TC exposure in countries where child marriage remains a threat to health and well-being, more locally focused analyses will be necessary to investigate the effects we observe in this small subset of settings, as well as potential effects that may be overlooked or obscured in broader national analyses. Further work will also be required in these settings to examine the role of potential factors that increase vulnerability, such as the direction of marriage payments.

A key strength of this study is its use of a large, multi-country dataset spanning 32 years, allowing for a comprehensive assessment of the relationship between TCs and child marriage across diverse economic, social, and cultural contexts. By estimating effects for each country rather than assuming a uniform relationship across all settings, we better account for the role of social norms, legal frameworks, and resilience to extreme weather. Using a distributed lag model further strengthens our approach by capturing the potentially overlapping effects of multiple TCs over time, providing a robust estimate of how child marriage rates evolve in the aftermath of these events. This study also substantially expands the geographic scope of prior research on the topic [37] and offers a more comprehensive understanding of the complex relationship between climate-sensitive events and child marriage.

Our study has several limitations that should be considered in future work. First, we use wind speed as a proxy for TC exposure. While wind speed is a widely used measure of TC intensity, it does not capture other important dimensions of TC impacts, such as flooding and storm surges. A consistent dataset linking cyclone-specific rainfall, storm surge, and flood inundation at the resolution our design requires is not currently available across our study countries, and is a priority for future work. However, given that extreme rainfall is often highly correlated with wind speeds [8], wind speed is likely a strong proxy for the majority of these secondary impacts. Second, our analysis assumed that no other external shocks (such as civil conflicts) systematically coincided with the occurrence of TCs during the study period. Given the widespread occurrence of TCs in many of the analysed countries, we consider the potential for this bias to be minimal. Third, given the lack of migration history data within four of the study countries, we were unable to determine the extent to which migration influenced the results in these countries. We encourage caution when interpreting for these countries. Fourth, our estimates capture the short-run effects of cyclones, up to three years after landfall. We cannot rule out longer-run or cumulative effects operating over greater time horizons, which are difficult to distinguish from other slow-moving structural changes unrelated to cyclone exposure. Finally, we were also unable to examine specific mediating economic mechanisms (such as dowry payments or cyclone-induced changes in household income and assets) because DHS does not record marriage payments and measures household wealth only at the time of the survey. Answering such questions in a cross-country context will require consistent collection of new data on marriage payments and household economic conditions over time.

Climate change will likely have profound and far-reaching effects on vulnerable populations. Our results add to the growing evidence suggesting that, though TCs may influence child marriage in some local settings, they are not a dominant driver of child marriage globally. As climate change accelerates, it will be essential to identify which impacts warrant the most significant intervention. Understanding these dynamics will be crucial for designing evidence-based policies that effectively support vulnerable populations in a changing climate.

## Data Availability

The data that support the findings of this study are openly available at the following URL/DOI: https://dhsprogram.com/ [38]; https://doi.org/10.5880/pik.2017.008 [53]. Supplementary data 1 available at https://doi.org/10.1088/2752-5309/aead12/data1.

## Data and code availability

All code needed to replicate this work is available at https://github.com/annapal/cyclone-marriage.
